# Menopausal status and the risk of lung cancer in women: A PRISMA-compliant meta-analysis

**DOI:** 10.1097/MD.0000000000007065

**Published:** 2017-06-30

**Authors:** Lingfeng Min, Fang Wang, Sudong Liang, Junjun Yang, Xingxiang Xu

**Affiliations:** a Department of Respiration, Clinical Medical School of Yangzhou University, Subei People's Hospital of Jiangsu Province, Yangzhou; b Department of Urology, Taizhou People's Hospital, Taizhou, Jiangsu, China.

**Keywords:** lung cancer, menopause, meta-analysis, reproductive, risk

## Abstract

**Background:**

Quantification of the association between menopausal status and risk of lung cancer is inconsistent. We carried out a meta-analysis of available studies to examine this issue.

**Methods:**

Relevant articles were identified by searching *PudMed* and *Embase* databases. Reference lists from selected papers were also reviewed. A random-effect model was used to calculate summary odds ratios (OR) and relative risk (RR) with 95% confidence interval (CI). Publication bias was estimated using Egger regression asymmetry test.

**Results:**

Eight eligible studies, including 5 case–control studies and 3 cohort studies, provided data for meta-analysis. Postmenopausal women had a statistically significant increased risk of lung cancer in all included studies (RR = 1.44, 95% CI: 1.12–1.85) and cohort studies (RR = 1.39, 95% CI: 1.05–1.86), but not in case–control studies (OR = 1.46, 95% CI: 0.95–2.24).

**Conclusions:**

Overall, there was evidence that postmenopause is related to increased lung cancer risk. However, studies have produced slightly heterogeneous results (*I*^2^ = 38.40%). To obtain a better indication of relationship, well-designed large prospective studies are required.

## 1 Introduction

Lung cancer is one of the most common cancers in the world and the leading cause of cancer mortality among women in the United States (US).^[1,2]^ In 2015, an estimated 105,590 women in the US were newly diagnosed with lung cancer and 71,660 died from this disease.^[2]^ The incidence of lung cancer is increasing in women, in contrast to lung cancer incidence rates in men, which have decreased or stabilized in most countries.^[3,4]^ Although cigarette smoking is a strong risk factor for lung cancer in women, approximately 10% to 15% of cases occur among never smokers,^[5]^ suggesting other independent factors play an important role in its etiology.

Numerous recent observational studies of female menopausal type and risk of lung cancer have provided only weak evidence of the role of menopausal type in lung cancer etiology, and results from menopausal studies have been inconsistent.^[6,7]^ Numerous observational findings have examined the relationship between lung cancer and menopausal types that include premenopause, natural postmenopause, non-natural menopause (i.e., surgical, or due to radiation or chemotherapy), and unknown types.^[6,8]^ However, results have been far from consistent across studies. To further investigate the association between menopausal status and risk of lung cancer, we conducted a meta-analysis of these studies.

## 2 Methods

### 2.1 Search strategy

This meta-analysis does not involve patients, and thus does not require institutional review board approval. We conducted a literature search of the *PubMed* and *Embase* databases for relevant published studies before October 2015. No language restrictions were applied, and the following search strategy was used: (“reproductive” or “menopause”) and (“lung” or “pulmonary”) and (“cancer” or “neoplasm” or “carcinoma”). We also perused the references of retrieved articles to identify additional potentially relevant articles.

### 2.2 Eligibility criteria

The following criteria were used to include relevant studies for meta-analysis: the study design was an original case–control or cohort study evaluating the relationship between menopausal type (pre- or postmenopause) and lung cancer; the outcome of interest was lung cancer incidence; odds ratio (OR) or relative risk (RR) estimates with 95% confidence interval (CI) were used, with adjustments for potential confounding factors, if applicable. If studies with the same or partially overlapping population were published in more than one study, we considered the most recent or comprehensive study.

### 2.3 Data extraction and exposure assessment

The following information was extracted from each eligible study: the first author's last name, year of publication, country of study, design, number of cases, menopausal status (pre- or postmenopause, natural or non-natural, that is, surgical, or due to radiation or chemotherapy, or unknown types) of the participants, smoking status, RR or OR, 95% CI, exposure assessment, and adjusted factors. Adjusted and unadjusted (when available) RR estimates (comparing participant groups defined by menopausal status) and their variance (or sufficient statistics to calculate that variance) were extracted. ORs were treated as RRs. We extracted the ORs and RRs that were adjusted for the greatest number of potential confounders.

In the 8 studies women were asked their menopausal status, for example, whether they were pre- or postmenopause, age at menopause, and type of menopause (natural, or induced by surgery, radiation, or chemotherapy). For surgical menopause, they were asked whether they had a hysterectomy with or without oophorectomy and number of ovaries removed. Participants were also asked their smoking status (former, current, never), and their environmental tobacco smoke exposure at home (during childhood and adulthood) and in the workplace. To reduce reporting bias, the studies did not reveal interviewer and participant interests, or inform the status of the participants to the interviewers.

### 2.4 Evidence synthesis

Studies gave RR and CI for at least 3 exposure categories (natural, surgical, and other), each compared with a single unexposed group (postmenopause). However, we needed the overall RR and CI for exposure versus no exposure. Relative risk estimation (RREst) is a spreadsheet program that manipulates nonindependent RRs^[9]^ and CIs to give the RR and CI of a comparison different from those provided. We used this method to estimate the relationship between subjects and exposure categories. The RR and CI for the specified contrast are calculated by combining the estimated numbers of subjects into baseline (i.e., specifying which of the available categories comprise the baseline) and comparison groups. The program also estimates heterogeneity and trend coefficients among the selected categories, and can be used for case–control and prospective studies.

### 2.5 Statistical analysis

We used the DerSimonian and Laird random-effects model to estimate an RR summary considering both within- and between-study variation.^[10]^ Statistical heterogeneity among studies was assessed using Cochrane *Q* test at the *P* < .05 level of significance and the *I*^2^ statistic.^[11]^ Subgroups were analyzed by study design and geographical area. We also performed a sensitivity analysis by omitting one study before pooling study-specific RRs to examine the influence of individual studies. We used Begg funnel plots and Egger linear regression test to assess publication bias.^[12,13]^ Statistical analyses were done using STATA Statistical Software, version 12.0 (Stata Corp, College Station, TX).

## 3 Results

### 3.1 Literature search

**Figure 1:**
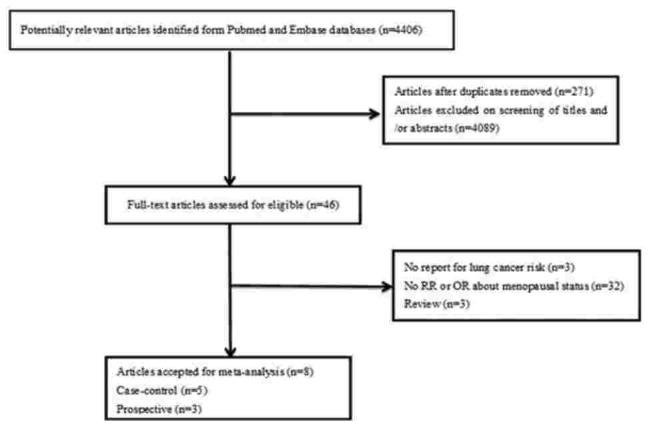
Flowchart of the selection of studies for the association between menopause and reproductive and lung cancer risk among women.

We searched *PubMed* and *Embase* databases and identified 4406 articles (*PubMed*: 3421, *Embase*: 985). A total of 271 duplicated articles and 4089 articles were removed at first screening of titles and abstracts. Full-text copies of the remaining 46 potentially eligible publications were obtained. Of these, 38 articles were excluded because they did not meet the following criteria: result was not lung cancer incidence (n = 3), no RR or OR for menopausal status (n = 32), and review (n = 3). The remaining 8 studies were included for meta-analysis (Fig. 1).

### 3.2 Study characteristics

Table 1 lists the basic characteristics of the 8 included studies. Five studies had a case–control design^[14–18]^ and 3 had a cohort design.^[19–21]^ Trials originated from the US (n = 2),^[18,19]^ Germany (n = 1),^[15]^ Japan (n = 2),^[16,20]^ China (n = 1),^[21]^ Canada (n = 1),^[14]^ and Italy (n = 1).^[17]^ Total sample size of the cohort studies was 383,391 subjects with 1197 lung cancer events; the total lung cancer cases for the case–control studies were 2411, and control cases in the case–control studies totaled 4499.

**Table 1:**
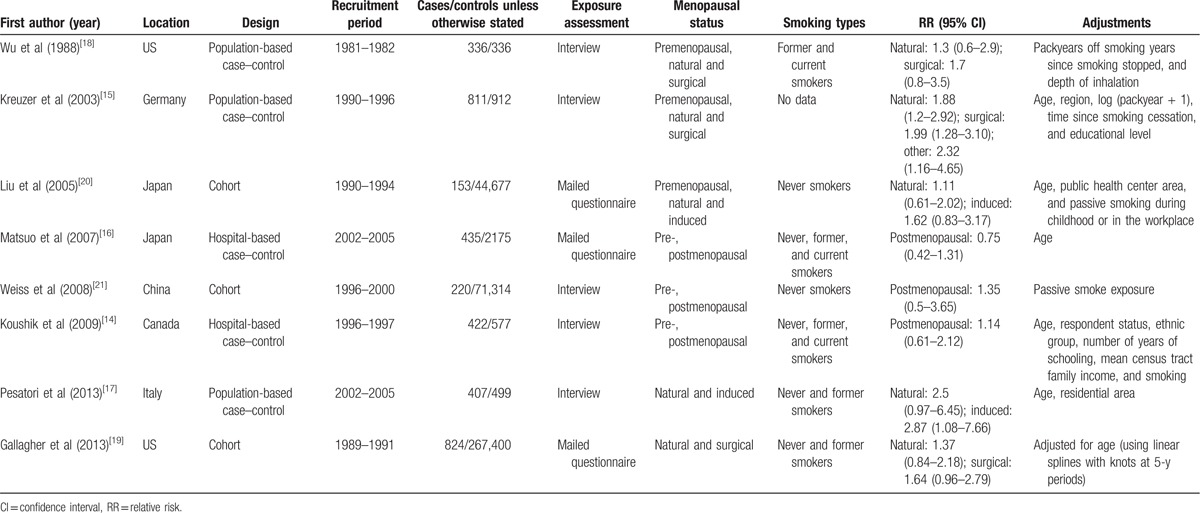
Characteristics of studies on lung cancer and menopausal status.

### 3.3 Evidence synthesis

We used the RREst program to enter each category description, and values/estimates for number of subjects, RR, and lower/upper 95% CI categories, to obtain specified contrast results. As an example, data for Wu et al included natural menopause (RR = 1.3, 95% CI: 0.6–2.9) and surgical menopause (RR = 1.7, 95% CI: 0.8–3.5) for which the program provided new data (RR = 1.52, 95% CI: 0.77–3.01). Using the same process provided data for Kreuzer et al (RR = 1.98, 95% CI: 1.37–2.86), Liu et al (RR = 1.27, 95% CI: 0.74–2.18), Pesatori et al (RR = 2.67, 95% CI: 1.28–5.59), and Gallagher et al (RR = 1.46, 95% CI: 4.02–2.1). These results were combined with the data for Matsuo et al, Koushik et al, and Weiss et al for the STATA statistical analyses.

### 3.4 Meta-analysis

**Figure 2:**
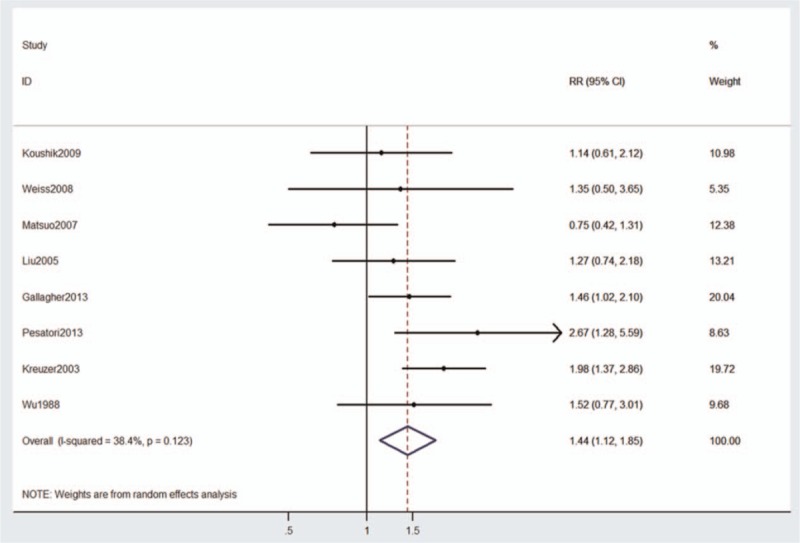
Risk estimates for the association between menopausal status and lung cancer risk in 8 studies and case–control and cohort study subgroups.

Overall risk estimates for lung cancer were elevated among postmenopausal women compared with premenopausal women (Fig. 2; RR = 1.44, 95% CI: 1.12–1.85), with no significant heterogeneity across studies (*P* = .123, *I*^2^ = 38.4%). A statistically significant 44% increased lung cancer risk was found to be associated with postmenopausal women. When stratified by study design, we found that postmenopause was associated with a statistically significant 39% increased lung cancer risk in cohort studies (RR = 1.39, 95% CI: 1.05–1.86), but not in case–control studies (OR = 1.46, 95% CI: 0.95–2.24).

**Figure 3:**
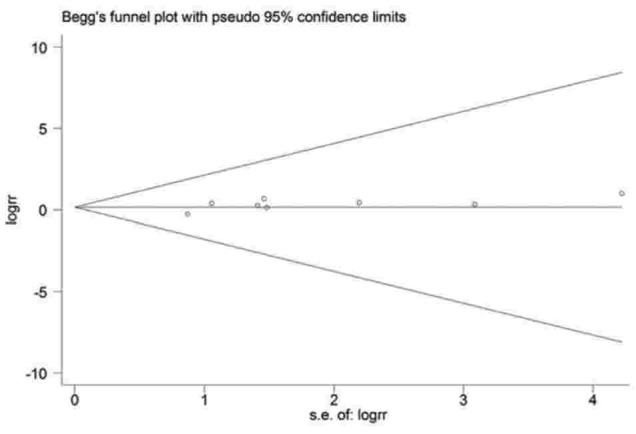
Funnel plot of 8 studies on the association between menopausal status and lung cancer risk.

We performed subgroup analyses by study design, study geographical area, assessment of exposure, adjusted for confounders, smoking, age, and smoking and age (Table 2). Postmenopause had a significant association with the risk of lung cancer (RR = 1.81, 95% CI: 1.19–2.75) in Europe, but not in Asia (RR = 1.03, 95% CI: 0.71–1.50). The summary RR estimate was increased slightly for the US (RR = 1.47, 95% CI: 1.07–2.03). A subgroup analysis of the assessment method of postmenopause was also performed. A statistically significant association was observed among studies using interviews (RR = 1.75, 95% CI: 1.35–2.27), but not among studies using mailed questionnaire techniques (RR = 1.17, 95% CI: 0.80–1.71). A statistically significant association was observed when we adjusted for age (RR = 1.43, 95% CI: 1.04–1.95), smoking (RR = 1.56, 95% CI: 1.22–2.00), and age and smoking (RR = 1.52, 95% CI: 1.06–2.17). There was no statistically significant heterogeneity in most subgroups. No indication of publication bias was observed form either with the Egger test (*P* = .142; Fig. 3) or Begg test (*P* = .711).

**Table 2:**
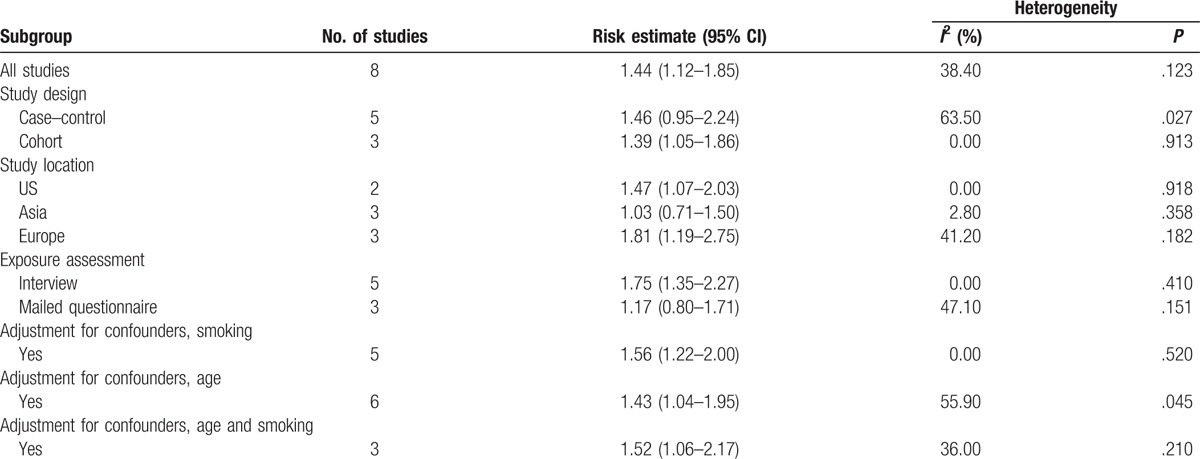
Summary relative risks for the association between menopausal status and lung cancer risk according to study design and geographical area.

## 4 Discussion

As far as we know, this is the first meta-analysis evaluating the relationship between menopausal status and lung cancer risk. In the extensive search, only 8 studies (5 case–control, 3 cohort) that met the inclusion criteria, although the total number of subjects was considerable (n = 390,301). Control study analysis suggested that postmenopausal women were at a higher risk of developing lung cancer than premenopausal women, but we failed to demonstrate this with the case–control studies. There was statistically significant heterogeneity of the 5 case–control studies (*I*^2^ = 63.50%); however, results from the 3 cohort studies were null. Case–control studies are more susceptible to recall and selection bias than cohort studies and the results from cohort studies may be more reliable. In subgroup analysis by study location, we found that postmenopause was associated with increased lung cancer risk in the US and Europe, but not in Asia, suggesting that ethnic genetic background and lifestyle differences should be considered. When we stratified the analysis by assessment of exposure, a significant association was found among studies that used interviews but not among studies that used mailed questionnaires. This contrast may be due to the differing techniques or chance alone.

There are several potential limitations. First, primary data were unavailable for most of the studies and additional adjustments for potentially important covariates could not be performed. Second, studies used different scales to evaluate menopausal status, so we could not analyze the risk of lung cancer in women who underwent natural or surgical menopause compared with those who had not undergone menopause. Differences in statistically adjusted RRs across studies may account for some unexplained heterogeneity. Third, the result may be influenced by age and smoking because cigarette smoke exposure is already established as an independent risk factor for developing lung cancer, but when we adjust for smoking and age, the result has not changed. Finally, we cannot preclude the possibility that other unpublished studies may have been missed despite extensive literature search in our meta-analysis. The potential publication bias may have been because studies with null effects were less easily published than those with positive effects, which made it different for us to obtain, although Egger test or Begg test did not reveal the present of publication bias.

A potential confounder is the inability to determine the role of natural or surgical menopause in lung cancer, as these data were incomplete. Despite its limitations, the study represents a possible connection between menopausal factors and lung cancer, providing direction for further studies of the role of menopause in the diagnosis, treatment, and survival of women with lung cancer.

In conclusion, current data indicate that menopausal status may increase the risk of lung cancer in women. The mechanisms involved are likely to be complex. Additional studies are warranted to extend our findings and to clarify the unknown mechanisms. Further studies are needed to investigate the effect of natural and surgical menopause on lung cancer risk and which type of lung cancer. The number of epidemiological studies was small in our meta-analysis; further well-designed, large prospective studies are needed to elucidate this relationship.
